# Progress of Surgery

**Published:** 1901-03-09

**Authors:** 


### Progress of Surgery.

### GENITO-URINARY AND RECTAL.

**Misplaced and Undescended Testicle.**—Thomas Annandale has contributed a paper on this subject.[1] He alludes to the three situations in which a misplaced testicle may be found—viz., in the perineum, at the saphenous opening, and in front of the pubes. When a testicle remains out of its natural position it degenerates and its structure alters, so that in time its functions are destroyed, and this is the case also when the testicle is retained in the abdomen or inguinal canal. Joseph Griffiths found that dogs' testicles quickly degenerated when returned into the abdominal cavity. Annandale believes that in 1879 he recorded the first case in which a testicle misplaced in the perineum was successfully transplanted into the scrotum. He attributes the success to certain details in the operation, which details he has also ever since successfully employed in dealing with undescended testicle. The incision exposes the spermatic cord and its junction with the testicle. The cord and testicle are drawn out, and all attachments abnormally fixing the latter divided. The affected side of the scrotum is opened up with the finger and the testicle placed in it. Two sutures are passed through those portions of the gubernaculum still attached to the testicle and through the base of the scrotum. The opening through which the testicle has passed into its abnormal situation is carefully stitched up with buried sutures. In the above case the testicle was found to have remained in its proper position after several years. The operation is to be urged and carried out in early childhood, for if successful it gives the testicle a chance of developing properly. It is also recommended that an attempt should be made in childhood to bring down a testicle which has appeared outside the abdominal cavity, but has not passed properly down into the scrotum, for the organ will not develop fully unless it is placed and retained in the scrotum. Such testicles are usually small, and more or less atrophied, but the operation gives them their only chance of developing. The indication for operation is stronger when both testicles have descended imperfectly. In adults, if an undescended testicle complicates the operation for the radical cure of a hernia Annandale excises it, because he considers it of no use, and the radical cure can be performed more satisfactorily if it is removed, but in young people he tries to bring down the testicle, especially if the other one is not properly placed. In his last four cases he has added another detail to the operation, that of passing the fixation sutures not only through the scrotum and remains of the gubernaculum, but also through the skin of the inner side of the thigh. Notes are then given of two cases of congenital deficiency of the testicle. In both swellings like small testicles were felt near the groin on the left side, and the corresponding portion of the scrotum was imperfectly developed. These swellings, after removal, were found to be epididymes only, the testicles themselves being absent.

**Tumours of the Testicle.**—James Pendersen, of New York contributes a paper on sarcoma of the testicle.[2] He first points out the similarity between sarcoma and carcinoma of the testicle in clinical history, ætiology, development, and malignancy. The two conditions are practically indistinguishable without microscopical examination, and even then confusion may arise. Sarcoma of the testicle may occur at all ages, even infancy is not exempt; and sometimes the small round-celled variety is congenital. Usually the growth is unilateral. Though the ætiological importance of traumatism is disputed, the balance of evidence supports the view that trauma is a true cause of the production of these tumours. Kocher is quoted as having observed six cases in which tumefaction of the gland followed immediately upon a blow. Lagrange performed orchidectomy three months after a blow and found a carcinoma present. It is owing to the injuries to which they are exposed that undescended or misplaced testicles are prone to develop malignant disease. Adenomata, enchondromata and cystic degeneration of the testicle are said to often end malignantly, but syphilitic orchitis and gonorrhœal epididymitis probably do not. The sarcoma rarely developes first in the epididymis, and if it does so it soon invades the body of the testicle. Soft areas due to the formation of cysts frequently form in the swelling. The skin may give way and fungus tufts protrude. The inguinal glands begin to enlarge when the skin is involved. Progression along the spermatic cord is slow. Death with metastases results in from one to two years. The epididymitis, which occurs in late syphilis, is mentioned as simulating malignant disease so closely that only anti-syphilitic treatment will clear up the diagnosis. The same applies to the interstitial form of syphilitic orchitis. The absence of pain is pointed to as being characteristic of the syphilitic affections. Generally speaking carcinoma is said to be more painful than sarcoma, and to form a softer tumour with less tendency to the formation of cysts and cartilaginous plates, and to more rapidly infect the inguinal and pelvic glands. A case of sarcoma is then recorded. The patient was aged twenty-seven, and the affected testicle had been driven through the inguinal canal fourteen years before by a severe blow from a base-ball, and the organ had never descended again. The growth formed a permanent, rounded tumour in the hypograstic and left inguinal regions. The primary growth was removed, but a secondary retro-peritoneal extension had to be left. Microscopically the tumour was found to be a large round-celled sarcoma devoid of any remnant of testicular tissue. T. Vincent Jackson, of Wolverhampton,[3] records an interesting case of testicular tumour. The patient was forty years of age, and had noticed the swelling seven or eight months. Its weight caused lumbar pain. The organ when removed weighed over a pound. A fortnight after the operation, the wound having healed and the patient allowed to get up, hæmoptysis suddenly set in. The presence of tubercle was negatived by every diagnostic test, including the injection of Koch's tuberculin. Two months later the man died from hæmoptysis. The structure of the tumour was reported by Mr. J. H. Targett, of the Clinical Research Association, to be composite and of congenital origin. It contained nodules of cartilage, bands of unstriped muscle, and tubulæ lined with ciliated epithelium. Also groups of giant cells were present, resembling those of tubercle, as though the tumour had been infected with that disease, as tumours are liable to be. The term chondro-carcinoma was said to be sometimes applied to this variety of growth.

**Operations for Hypospadias.**—R. Hamilton Russell, of Melbourne,[4] describes an operation for severe forms of hypospadias. He points out that such meagre results have hitherto followed these operations in bad perineoscrotal hypospadias that many surgeons consider them not worth attempting. It has even been suggested that it would be humane and wise to perform castration in childhood, as it is so hopeless to provide the patient with an effective sexual organ. The two objects of the operation are to render the penis effectual as a sexual organ and to enable the subject to perform micturition in masculine fashion. The only method devised hitherto which has had substantial success is said to be that of Duplay. Russell recommends two operations in place of the three of Duplay. The main features of the first operation are as follows:—The penis is first liberated by an incision through the frænum which binds down the glans. This incision is carried round the penis circularly near the corona. The dense fibrous bands holding down the penis are divided. Then the glans is perforated and a capacious channel made through it. Next on each side an incision is made along the under surface of the penis parallel to the edges of the raw surface already made, and the incisions are united anteriorly by another carried over the dorsum of the penis parallel to the first cut carried round the dorsum and a short distance behind it. By these incisions a strip of prepuce will be marked out which surrounds the penis like a clergyman's stole. This is detached everywhere except at its extremities near the root of the penis, and is slipped over the end of the penis just as a stole is removed. The loop is seized in a sinus forceps and pulled through the channel in the glans. The redundant portion is cut off, and the two lateral portions fixed in position by one or two sutures at the meatus. A skin-covered lining for the new urethra from the perinæum to the glans is thus provided. The edges of the skin wound beneath the penis are sutured so as to cover the new urethra, each suture taking up the two edges of the new urethral lining in Lembert fashion as well as the edges of the wound. The second operation consists in performing suprapubic cystotomy and closing the perineal urethra. The latter is difficult to accomplish. The incisions are made on each side of the groove lined with mucous membrane, which exists in front of the perineal opening, and the perineal skin is undermined slightly on each side and the flaps united in the middle line. One successful case is recorded. Carl Beck,[5] of New York, has written on the operation he devised some years ago for hypospadias. He states that he found a hundred married hypospadiacs, none of whom had had children. A consideration of the normal extensibility of the penis during erection led him to evolve his operation. The elasticity of the mucous membrane which permitted such extension permits also that a long area may be covered by the urethra if it be dissected up and stretched. His operation briefly consists in exposing and dissecting up the urethra, together with its corpus cavernosum, from the abnormal orifice back to the posterior third of the pendulous portion of the penis. The glans is then tunnelled and the urethra stretched and made to lie in the tunnel and sutured to the free margin of the new meatus. The skin edges are then united below.

[1] Brit. Med. Jour., Dec. 1900. [2] The Post Gradu te, Aug. 1900. [3] Lancet, Dec. 1900. [4] Brit. Med. Jour., Nov. 1900. [5] Med. Rec., Oct. 1900.

